# Assessment of the alpha 7 nicotinic acetylcholine receptor as an imaging marker of cardiac repair-associated processes using NS14490

**DOI:** 10.1186/s13550-023-01058-2

**Published:** 2024-01-11

**Authors:** Victoria J. M. Reid, Wesley K. X. McLoughlin, Kalyani Pandya, Holly Stott, Monika Iškauskienė, Algirdas Šačkus, Judit A. Marti, Dominic Kurian, Thomas M. Wishart, Christophe Lucatelli, Dan Peters, Gillian A. Gray, Andrew H. Baker, David E. Newby, Patrick W. F. Hadoke, Adriana A. S. Tavares, Mark G. MacAskill

**Affiliations:** 1 Centre for Cardiovascular Science, The University of Edinburgh, Edinburgh, UK; 2 Edinburgh Imaging, The University of Edinburgh, Edinburgh, UK; 3 Department of Organic Chemistry, Kaunas University of Technology, Kaunas, Lithuania; 4 Proteomics and Metabolomics Facility, The Roslin Institute, University of Edinburgh, Edinburgh, UK; 5 DanPET AB, Malmo, Sweden; 6 Neurobiology Research Unit, Copenhagen University Hospital Rigshospitalet, Copenhagen, Denmark

**Keywords:** Alpha 7 nicotinic acetylcholine receptor, Imaging, Myocardial Infarction, Cardiac Repair

## Abstract

**Background:**

Cardiac repair and remodeling following myocardial infarction (MI) is a multifactorial process involving pro-reparative inflammation, angiogenesis and fibrosis. Noninvasive imaging using a radiotracer targeting these processes could be used to elucidate cardiac wound healing mechanisms. The alpha7 nicotinic acetylcholine receptor (ɑ7nAChR) stimulates pro-reparative macrophage activity and angiogenesis, making it a potential imaging biomarker in this context. We investigated this by assessing in vitro cellular expression of ɑ7nAChR, and by using a tritiated version of the PET radiotracer [^18^F]NS14490 in tissue autoradiography studies.

**Results:**

ɑ7nAChR expression in human monocyte-derived macrophages and vascular cells showed the highest relative expression was within macrophages, but only endothelial cells exhibited a proliferation and hypoxia-driven increase in expression. Using a mouse model of inflammatory angiogenesis following sponge implantation, specific binding of [^3^H]NS14490 increased from 3.6 ± 0.2 µCi/g at day 3 post-implantation to 4.9 ± 0.2 µCi/g at day 7 (n = 4, *P* < 0.01), followed by a reduction at days 14 and 21. This peak matched the onset of vessel formation, macrophage infiltration and sponge fibrovascular encapsulation. In a rat MI model, specific binding of [^3^H]NS14490 was low in sham and remote MI myocardium. Specific binding within the infarct increased from day 14 post-MI (33.8 ± 14.1 µCi/g, *P* ≤ 0.01 versus sham), peaking at day 28 (48.9 ± 5.1 µCi/g, *P* ≤ 0.0001 versus sham). Histological and proteomic profiling of ɑ7nAChR positive tissue revealed strong associations between ɑ7nAChR and extracellular matrix deposition, and rat cardiac fibroblasts expressed ɑ7nAChR protein under normoxic and hypoxic conditions.

**Conclusion:**

ɑ7nAChR is highly expressed in human macrophages and showed proliferation and hypoxia-driven expression in human endothelial cells. While NS14490 imaging displays a pattern that coincides with vessel formation, macrophage infiltration and fibrovascular encapsulation in the sponge model, this is not the case in the MI model where the ɑ7nAChR imaging signal was strongly associated with extracellular matrix deposition which could be explained by ɑ7nAChR expression in fibroblasts. Overall, these findings support the involvement of ɑ7nAChR across several processes central to cardiac repair, with fibrosis most closely associated with ɑ7nAChR following MI.

**Supplementary Information:**

The online version contains supplementary material available at 10.1186/s13550-023-01058-2.

## Introduction

Wound healing in response to injury is a complex and multifactorial response, which leads to tissue remodeling and repair. Following myocardial infarction (MI), the dynamics of the cellular responses leading to wound healing have been demonstrated through detailed preclinical and clinical studies [1, 2]. From these studies, it is clear that progression of the wound repair process is an important determinant of long-term remodeling and loss of cardiac function leading to the development of heart failure. As yet there remains no reliable means of noninvasively assessing the processes involved in cardiac wound healing, or directly determining the impact of therapeutic intervention. Cardiac repair involves a complex interplay between a multitude of cell types such as immune cells, endothelial cells and fibroblasts. This coalition of cells drive the processes of inflammation, angiogenesis and extracellular matrix reorganization which are central to the cardiac healing response [3]. The alpha7 nicotinic acetylcholine receptor (ɑ7nAChR) plays a role in both angiogenesis and promotion of a pro-reparative macrophage phenotype during wound repair [4, 5]. In addition, ɑ7nAChR has been shown to be central to cardiac repair with blockade leading to increased adverse remodeling [6] while stimulation has cardioprotective effects [7, 8]. As such, we set out to investigate the suitability of this target as an imaging biomarker which reflects the interlinked processes which are essential to cardiac wound healing.

The ɑ7nAChR is a pentameric ligand-gated ion channel that is expressed by central and peripheral neuronal cells [9]. It is also expressed by peripheral non-neuronal cells, such as epithelial [10], endothelial [4], vascular smooth muscle [11] immune cells [12] and fibroblasts [13]. Peripheral activation of ɑ7nAChR by neuronal and non-neuronal acetylcholine release leads to anti-inflammatory regulation of the innate immune system and is one of the most well-defined actions of this receptor [5]. ɑ7nAChR is an essential regulator of inflammatory macrophage activity [12] and stimulates the polarization and survival of an anti-inflammatory M2-like macrophage phenotype [14]. These M2-polarized macrophages are essential for resolution and inflammation and promotion of pro-reparative activities including angiogenesis and fibrosis [15]. Although less explored, ɑ7nAChR activation in endothelial cells is also known to directly play a role in the stimulation of angiogenesis. ɑ7nAChR is the most abundant nicotinic acetylcholine receptor subtype within endothelial cells [4]. Activation or inhibition of this receptor has pro- and anti-angiogenic consequences, respectively, as demonstrated in vitro and in vivo [4, 16, 17]. Nicotine, a ligand of ɑ7nAChR, is a powerful promotor of angiogenesis via ɑ7nAChR-dependent means [18]. Given the role ɑ7nAChR plays in the above wound healing processes, it is likely that this receptor may also play a role in the other elements of cardiac repair such as fibroblast regulation of fibrosis. However, there is a lack of evidence to support this and as such this study initially focused on the inflammatory and angiogenic aspects of ɑ7nAChR activation.

We hypothesize that ɑ7nAChR can serve as an imaging target for assessment of angiogenesis and pro-reparative inflammation using the ɑ7nAChR selective agonist Positron Emission Tomography (PET) radiotracer [^18^F]NS14490 [19, 20]. We investigated the in vitro expression of this target in human macrophages, endothelial and vascular smooth muscle cells during proliferative and hypoxic conditions. In addition, we assessed this receptor’s relationship to inflammation and angiogenesis using the inflammation-driven angiogenic sponge implantation model with a tritium labeled version of NS14490 and autoradiography to measure ɑ7nAChR specific binding. Finally, we investigated the pattern of ɑ7nAChR expression during left ventricular remodeling following MI using tissue from a rat MI model.

## Methods

### Normoxic and hypoxic cell culture

Three different human cell types were cultured in this study. Primary human umbilical vein endothelial cells (HUVEC, Promocell, Germany) were cultured on 0.1% gelatin (Sigma, UK) using Endothelial Growth Medium 2 (Promocell, Germany) and used between passages 2–6. The monocytic cell line THP-1 (ECACC, UK Health Security Agency, UK) was cultured in suspension using RPMI supplemented with 10% fetal calf serum and 2 mM L-glutamine (Sigma, UK). Prior to investigation, THP-1 cells were differentiated into macrophages using 100 ng/ml of phorbol 12-myristate 13-acetate (PMA, Sigma, UK) treatment for 72 h. This leads to a non-polarized macrophage phenotype with a negligible level of cell proliferation. Primary human coronary artery smooth muscle cells (CVSMC, Promocell, Germany) were cultured using Smooth Muscle Cell Growth Medium 2 (Promocell, Germany) and used between passages 2–6. All cells were maintained at 37 °C, 5% CO_2_ and 100% humidity under normoxic conditions (atmospheric oxygen). The endothelial and vascular smooth muscle cell’s basal, non-proliferative (quiescent), state was achieved though contact inhibition by allowing the cells to grow to 100% confluence. Sub-confluent cultures were exposed to hypoxia (1% O_2_) over a time course (0-16 h). Cells were then lysed using RIPA buffer system (Santa Cruz Biotechnology, USA), and the protein was stored at -20 °C.

To assess the presence of ɑ7nAChR signaling in cardiac fibroblasts of the rat heart, rat cardiac fibroblasts where isolated and cultured for assessment of ɑ7nAChR expression consistent with how the human cell cultures were investigated. Experiments were authorized by the local University of Edinburgh Animal Welfare and Ethical Review Committee and in accordance with the Home Office Animals (Scientific Procedures) Act of 1986. Rat cardiac fibroblasts were isolated and maintainted as previously desribed [21–23]. Briefly, two male and two female Sprague–Dawley rats at 4 weeks of age were culled using an overdose of anesthetic. Prior to dissection, fur was shaved and skin was sterilized with ethanol, hearts were dissected using sterilized tools and placed in warmed DMEM/F-12 (Gibco, USA). Following dissection, pericardium and atria were removed using sterile tools and ventricles were weighed. Cardiac tissue was manually dissociated into 1-2mm^2^ pieces and each heart was transferred to a Miltenyi C tube (Miltenyi Biotec, Germany). The tissue was then digested using the enzyme mix from Multi Tissue Dissociation Kit 2 (Miltenyi Biotec, Germany) for 15 min at 37 °C and then further dissociated using the gentleMACS Dissociator (Miltenyi Biotec, Germany). These steps were repeated twice further. Samples were then resuspended in DMEM/F-12 with 20% FBS (Gibco, USA) and filtered using a 70-µm strainer. The cell suspension was centrifuged at 600 × g for 5 min, after which the supernatant was discarded before the pellet was resuspended in DMEM/15% FBS/1% Penstrep (Gibco, USA). The single-cell suspensions across the four hearts were pooled and then placed onto a poly-L-lysine coated T75 flask (Merck/Sigma-Aldrich, USA) and incubated for 2 h. Following incubation, the single-cell suspension was removed leaving the fibroblasts adhered to the coated flask. The isolated fibroblasts were maintained and expanded using DMEM/15% FBS/1% Penstrep. These cells were characterized by immunocytochemical analysis and found to be Vimentin^+^/PDGFRα^+^, heterogeneously positive for αSMA and CD45^−^/RECA-1^−^. Following cell expansion cells were seeded at a density of 700,000 cells per T25 and assed under the same conditions as the human vascular cells. These conditions were carried out in triplicate.

### Western blot analysis of α7nAChR

The protein content of lysed cells was quantified using the Bio-Rad Protein Assay which is based on the Bradford Method (Bio-Rad, USA). Protein was reduced and prepared for western analysis using the Bolt LDS sample buffer and Reducing agent, before 10 µg protein was loaded into each well of a 10% Bis–Tris Plus Bolt Gels (Thermo Fisher Scientific, USA). Gels were run for 60 min at 150 v using NP MES SDS running buffer (Thermo Fisher Scientific, USA) and transferred to Nitrocellulose membrane at 10 v for 60 min using NuPAGE transfer buffer (Thermo Fisher Scientific, USA). Membranes were blocked with 5% milk in TBST before overnight incubation with the primary antibodies at 4 °C. The anti-ɑ7nAChR was used at 1/300 (ab10096, Abcam), while anti-GAPDH was used at 1/10000 (ab9485, Abcam). Proteins were then detected using IRDye 800CW-labeled antibodies and visualized on the Odyssey system (LI-COR Biosciences, USA). ɑ7nAChR was assessed relative to the GAPDH protein loading control using Image Studio (LI-COR Biosciences, USA) which included blot background correction.

### Mouse sponge implantation model

Experiments were authorized by the local University of Edinburgh Animal Welfare and Ethical Review Committee and in accordance with the Home Office Animals (Scientific Procedures) Act of 1986. Twenty-four adult male C57Bl/6 J mice aged 8 − 10 weeks were housed under standard conditions of 12 h of light and 12 h of darkness, with food and water available ad libitum. The sponge implantation was carried out as previously described [24]. Briefly, anesthesia was induced and maintained with 1.5–2.5% isoflurane (0.5–3% in oxygen, 1 L/min) with body temperature maintained by a heated mat. Buprenorphine (0.05 mg/kg; Sogeval, France) was given subcutaneously for analgesia. The area around the scruff was shaved and disinfected with sterilizing iodine solution before a 1-cm incision was made into the skin between the shoulder blades. A subcutaneous tunnel was made using blunt forceps to lift the skin from the back of the body. The sponges (1.5 × 1 × 1 cm; Caligen Foam, Accrington, Lancashire, UK) were inserted into the mouse’s flank before closing the incision with 2 − 3 Michel wound clips. The animal was allowed to recover and closely monitored for signs of infection over the following week. Mice were culled at 3-, 7-, 14- and 21 days post-surgery, with metal clips being removed 10 days post-surgery once the wound had healed, where applicable. Excised sponges were fixed in 10% formalin (Sigma, UK) before paraffin tissue processing.

### Myocardial ischemia and reperfusion injury in rats

Experiments were authorized by the local University of Edinburgh Animal Welfare and Ethical Review Committee and in accordance with the Home Office Animals (Scientific Procedures) Act of 1986. Thirty-five adult male Sprague–Dawley rats aged 7–8 weeks underwent the surgery, n = 17 for the sham cohort and n = 18 for the MI cohort. The animals were housed under standard conditions of 12 h of light and 12 h of darkness, with food and water available ad libitum. Anesthesia was induced and maintained using isoflurane (0.5–3% in oxygen, 1 L/min) before buprenorphine (0.05 mg/kg; Sogeval, France) was administered preoperatively for analgesia. Tracheal intubation was achieved under direct vision, and ventilation was maintained with a rodent ventilator (model 683, Harvard Apparatus, USA) according to the manufacturer’s instructions. The skin was incised at the level of the left third and fourth ribs where the pectoral muscles were divided and retracted. A left lateral thoracotomy was then performed. With minimal handling, the pericardium was ruptured, the heart gently exteriorized from the thorax, and a silk 5–0 ligature was placed around the left anterior descending coronary artery just above the bifurcation of the first diagonal and maneuvered back into position. After 30 min, this ligature was removed to allow reperfusion and the wound was closed in three layers. The sham procedure omitted the coronary artery ligation step. Animals were recovered and extubated once spontaneous ventilation was established, housed at 30 °C for 24 h and given sterile sodium chloride fluid therapy (0.9%, 0.01 mL/g) subcutaneously in addition to another dose of buprenorphine. After 24 h, normal housing conditions were resumed. Rats were culled at 2, 7, 14 and 28 days post-surgery, and their hearts were collected and fixed in 10% formalin (Sigma, UK) before paraffin tissue processing.

### α7nAChR Autoradiography with [^3^H]NS14490

The tritiated version was utilized in this study due to the higher spatial resolution afforded by this isotope relative to ^18^F. Formalin-fixed, paraffin-embedded sponge and cardiac tissue were sectioned at 5 µm thickness onto glass slides, then dewaxed and rehydrated just prior to autoradiography. The slides were left to equilibrate in buffer (50 mM Tris–HCl, 4 mM CaCl2, 0.1% bovine serum albumin (BSA), 120 mM NaCl, 5 mM KCl, pH 7.4) for 30 min at room temperature before they were incubated with 50 nM [^3^H]NS14490 (Novandi Chemistry AB, Sweden) for 2 h at room temperature (total binding). Non-specific binding was determined in separate sections using 50 nM [^3^H]NS14490 with the addition of non-radioactively labeled NS14490 at 10 µM. Slides were washed twice in the assay buffer, followed by a dip in deionized H_2_O and left to dry before exposure to a BAS TR2040 imaging plate for 4 weeks. A set of tritium standards were incubated along with the slides and used to convert binding into µCi/g (ART-123A, ARC Inc., USA). The plates were imaged on an Amersham Typhoon system (Cytiva, USA) at 10 µM resolution using the 4000-sensitivity option. Image J was used for analysis with the represented images show as the “Fire” lookup table. Binding was converted to µCi/g before specific binding was calculated by subtracting the non-specific slide value from the total slide value. Regions of interest (ROI) were drawn on hematoxylin and eosin (H&E) stained serial sections and transposed into the autoradiography data. Where the average of an ROI had no specific binding, this region was given a value of 0.

### Histology and immunostaining

Serial 5-µm transverse paraffin-embedded sections were dewaxed and rehydrated through graded alcohols before staining. To visualize cellular and tissue structure, H&E staining was performed by incubation of rehydrated slides for 5 min in hematoxylin. The slides were then washed in 1% acid alcohol (5 s) and differentiated in Scott’s tap water substitute (10 s). Slides were then incubated in eosin (5 s) before thoroughly washing and dehydrating through graded alcohol. The slides were cleared in xylene and mounted using synthetic resin.

Serial slides were also stained for total collagen by with Picro Sirius Red through a 60-min incubation in the dye solution (ab150681, Abcam plc), followed by two washes in 0.5% acetic acid (3 s) and dehydrating through graded alcohol. The slides were cleared in xylene and mounted using synthetic resin.

Immunostaining was carried out on the mouse sponge tissue for the endothelial cell marker CD31 (AB28364, Abcam, UK), the vascular smooth muscle cell marker α-SMA (Sigma, USA) and the macrophage marker F4/80 (eBiosciences, USA). For CD31 and alpha smooth muscle actin (α-SMA) co-staining, slides were dewaxed and rehydrated before antigen retrieval in a pressure cooker using citrate buffer (pH 6). Slides were washed in 0.5% BSA in PBS at the beginning and between each antibody step. Blocking was performed for 1 h in 10% donkey serum and before incubation with the primary CD31 antibody (1/200) overnight at 4 °C. This was followed by incubation with the secondary Alexa 488 conjugated antibody (donkey anti-rabbit, 1/1000, Sigma, UK) and α-SMA antibody conjugated to Cy3 (rabbit anti-mouse, 1/1000). The nuclear counter stain DAPI was applied (1/1000) for 5 min before a final thorough wash and then mounting. For F4/80, slides were dewaxed and rehydrated before antigen retrieval was performed with trypsin (0.5 mg/mL, 10 min at 37˚C). Endogenous peroxidases were blocked with a 15-min H_2_O_2_ incubation (3%), and antibody blocking was achieved with the Vector impress blocking system (Vector Labs, USA). The F4/80 antibody was applied (1/300) for 30 min followed by an incubation with the ImmPRESS™ anti-rat Ig reagent for 30 min (Vector Labs, USA).

Immunostaining was also carried out on rat cardiac tissue for the monocyte lineage marker CD68 (Bio-Rad Laboratories, USA). Slides were dewaxed and rehydrated before antigen retrieval in a pressure cooker using Tris–EDTA buffer (pH 9.0). Endogenous peroxidases were blocked using 3% hydrogen peroxide for 15 min, followed by a PBS and blocking step in 10% donkey serum for 1 h. All subsequent incubations were performed at room temperature, with PBS washes carried out every step after the primary incubation. Slides were then incubated with the primary antibody at 1:100 in 1% donkey serum for one hour before incubation with the secondary donkey anti-mouse peroxidase at 1:750 (Jackson ImmunoResearch, USA) for one hour. The signal was then visualized using Tyramide amplification through incubation with Tyramide red working solution (TSA™-Plus Cyanine 3 System, PerkinElmer Inc. USA) at 1:50 for 3 min. Nuclei were counterstained with DAPI.

Brightfield and fluorescent images were scanned at 20 × magnification (Axioscan slide scanner, Zeiss, Germany, with Hitachi HV-F202SCL imaging device). All slides were processed at the same time to minimize variation in staining intensity. Quantification was performed using Image J with ROIs drawn on the H&E sections. To quantify total collagen, the % red area within the ROI was calculated. For quantification of fluorescent images, the mean intensity of the CD68 signal within the ROIs was calculated. These values were then plotted against the α7nAChR specific binding signal for the matching ROI.

### Cardiac proteomics and pathway analysis

Proteomics was carried out in pooled cardiac tissue, with 4–6 hearts used for each time point. Approximately 100 to 250 mg of tissue pooled samples were extracted with lysis buffer (5% sodium dodecyl sulfate (SDS) in 50 mM triethylammonium bicarbonate buffer (TEAB)) in a ratio of 1:10 (w/v) and homogenized with ceramics beads (CK Mix Precellys ceramics beads; Bertin Technologies, France). After beads beating, each vial was centrifuged 10,000 RCF for 10 min. Resulting supernatant was transferred into a new protein low binding vial, sonicated for 10 min (Bioruptor Picosonicator; Diagenode, Belgium) and centrifuged 10,000 RCF for 10 min. 500 µL of each clean supernatant were taken and protein concentration was determined using BCA assay (Thermo Scientific, USA); 30 µg of protein in 25 µL of lysis buffer was then reduced with 10 mM dithiothreitol (final concentration) for 1 h, 400 rpm at 37 °C and alkylated with 18.75 mM iodoacetamide for 35 min at room temperature in the dark. Reduced and alkylated supernatant were digested and washed with S-trap microcolumns (ProtiFi, USA) with a high recovery protocol as described by the suppliers, adding three extra chloroform:methanol (200 µL, 1:1, v/v) washes to remove the lipid content and using trypsin/Lys-C enzyme at an enzyme–substrate ratio of 1:20 for digestion. Peptides were eluted with 60 µL of 50 mM TEAB, followed by 60 µL of 0.1% formic acid and 60 µL of 50% acetonitrile (MeCN) in 0.1% formic acid. Eluted peptides were dried in a SPD2010 SpeedVac (Thermo Scientific, USA) and desalted using Hypersep C18 Spin tips (Thermo Scientific, USA) and finally eluted with 0.1% trifluoro acetic acid (TFA) in 60% MeCN and dried in a SpeedVac.

Purified peptides were separated over a 90 min gradient on an Aurora-25 cm column (IonOpticks, Australia) using a UltiMate RSLCnano LC System (Dionex, USA) coupled to a timsTOFfleX mass spectrometer (Bruker Daltonics, Germany) through a CaptiveSpray ionization source. The gradient was delivered at a flow rate of 200 nL/min, and washout was performed at 500 nL/min. The column temperature was set at 50 °C. For DDA-PASEF acquisition, the full scans were recorded from 100 to 1700 m/z spanning from 1.45 to 0.65 Vs/cm2 in the mobility (1/K0) dimension. Up to 10 PASEF MS/MS frames were performed on ion-mobility separated precursors, excluding singly charged ions which are fully segregated in the mobility dimension, with a threshold and target intensity of 1750 and 14,500 counts, respectively. Raw mass spectral data was processed using PEAKS Studio X-Pro Software (Bioinformatics Solutions Inc., Canada). Search was performed against UniProt rat reference proteome (Rattus norvegicus) sequence database containing 22,866 entries with MS1 precursor tolerance of 20 ppm and MS2 tolerance of 0.06 Da. Fully tryptic digestion allowing one missed cleavage, fixed modification of cystine (+ 57.02) and oxidation of methionine and deamination of asparagine and glutamine were specified as variable modification for database search. Peptide spectrum matches, peptides, and proteins were validated at a 1.0% FDR using the decoy hit distribution. Quantitative LFQ analysis was performed and with optional ID transfer enabled. Following investigation of several normalization approaches, protein expression relative to GAPDH was found to be the most appropriate by investigating other validated internal housekeeping markers (HPRT1 [25], Park7 [26]) and has been shown to be the most stable protein for rat cardiac tissue [25]. Missing data points were imputed, and statistical changes were calculated as previously described [27]. Differentially expressed up- and downregulated proteins were identified using a fold change cutoff of 2 and p < 0.05. These protein hits then underwent functional enrichment analysis on WebGestalt [28] using over-representation analysis and the following parameters: minimum number of IDs in the category = 5, maximum number of IDs in the category = 2000, FDR method = BH, significance level = FDR < 0.05.

### Statistical analysis

Other than proteomics, all statistical analysis was performed using Graphpad Prism version 9. All graphical results are displayed as the mean ± standard error of the mean (SEM). Normality analysis was based on D'Agostino-Pearson and Shapiro–Wilk, and the following statistical tests were used; Kruskal–Wallis test with post hoc Dunn’s, ordinary one-way ANOVA with post hoc Holm-Šidák test, ordinary one-way ANOVA with post hoc Dunnett’s, two-way ANOVA with post hoc Šidák’s test, Pearson correlation.

## Results

### Cellular α7nAChR expression in human macrophage and vascular cells shows an endothelial cell-specific hypoxia response

**Fig. 1:**
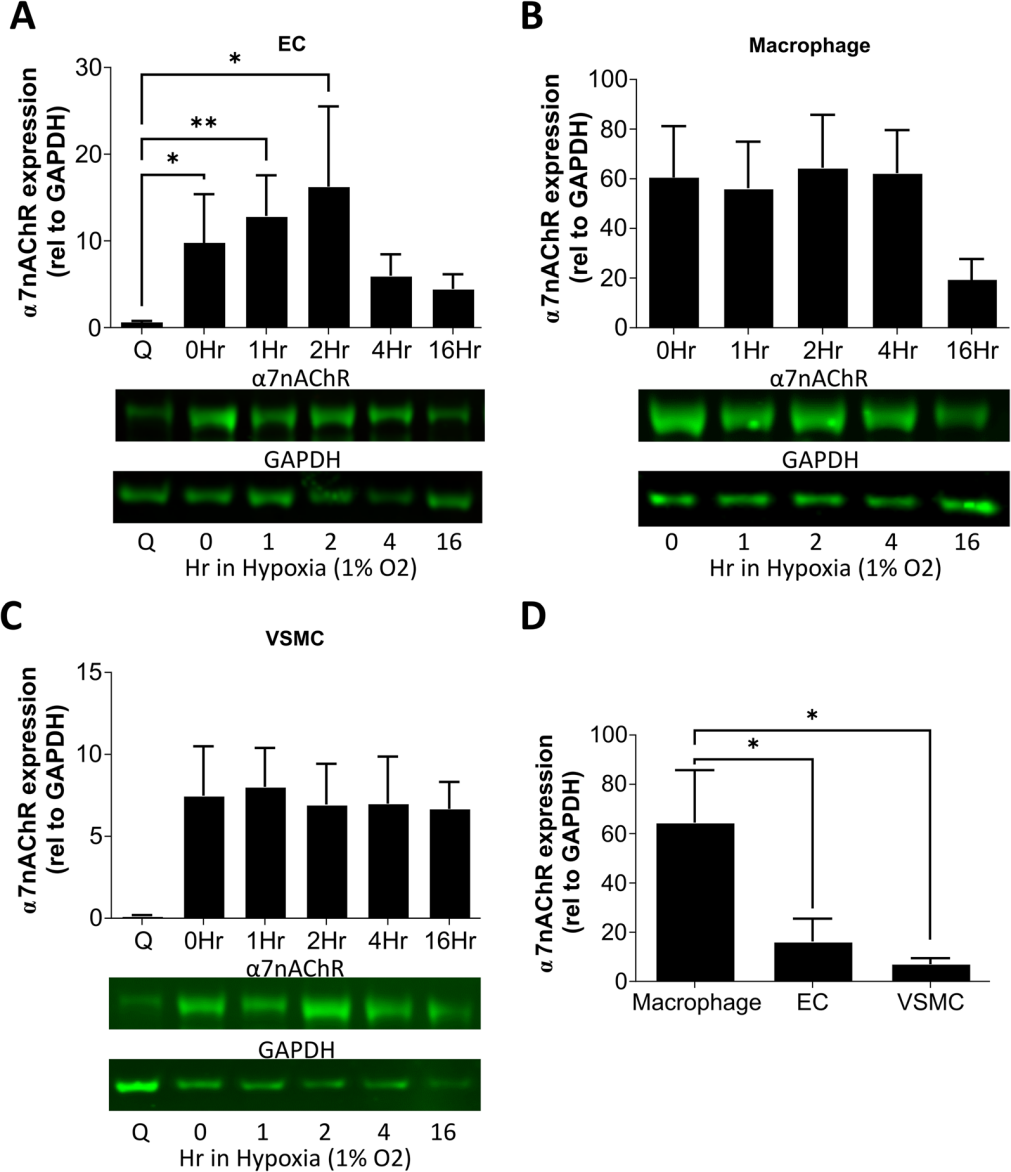
Analysis of α7nAChR protein in human cells associated with vascular regeneration and repair showing an endothelial-specific hypoxia response, and the highest α7nAChR expression relative to GAPDH within macrophages. **A** Western blot quantification of human endothelial cell (HUVEC) α7nAChR expression during quiescent conditions (Q), normoxic proliferating conditions (0Hr) and exposure to 1% hypoxia (1–16 h) in proliferating conditions. *N* = 6, * = *P* ≤ 0.05, ** = *P* ≤ 0.01 using a Kruskal–Wallis test with post hoc Dunn’s. **B** Western blot quantification of human macrophage (PMA-differentiated THP-1) α7nAChR expression during normoxic conditions and 1% hypoxia. These cells no longer proliferate following PMA differentiation, so no quiescent conditions were used, *N* = 4–5. **C** Western blot quantification of vascular smooth muscle cell (human coronary artery) α7nAChR expression during quiescent conditions (Q), normoxic proliferating conditions (0Hr) and exposure to 1% hypoxia in proliferating conditions, *N* = 4–5. **D** Direct comparison of α7nAChR expression across the cell types at 2 h in 1% hypoxic conditions, *N* = 5–6 and * = *P* ≤ 0.05 using ordinary one-way ANOVA with post hoc Holm-Šidák test. All results are shown as the mean ± SEM and example blots are shown

The response of α7nAChR in human endothelial cells, vascular smooth muscle cells and macrophages to normoxic and hypoxic conditions was investigated by western blot (Fig. 1, Additional file 1: Fig S1). Proliferative conditions (normoxia, full growth media) stimulated an increase in endothelial α7nAChR expression versus baseline quiescent conditions (normoxia, 100% confluent). Hypoxia also increased α7nAChR expression in endothelial cells, peaking after 2 h exposure to 1% O_2_ (Fig. 1A). Macrophages exhibited no change in α7nAChR expression in response to hypoxia (Fig. 1B). The effect of proliferative conditions on macrophage α7nAChR expression was not investigated as PMA differentiation of THP-1 monocytes results in a non-proliferative cell phenotype [29]. Vascular smooth muscle cells showed a trend (*P* = 0.069, Kruskal–Wallis test) toward increased α7nAChR expression during proliferation, but was increased further by hypoxia (Fig. 1C). Comparison of α7nAChR expression between the three cell types under proliferative and hypoxic conditions (2 h) revealed the highest relative α7nAChR expression was found in the macrophages, followed by the endothelial cells and vascular smooth muscle cells (Fig. 1D).

### α7nAChR signal in a murine inflammatory angiogenesis sponge implantation model peaks at the onset of new vessel formation and macrophage infiltration

**Fig. 2:**
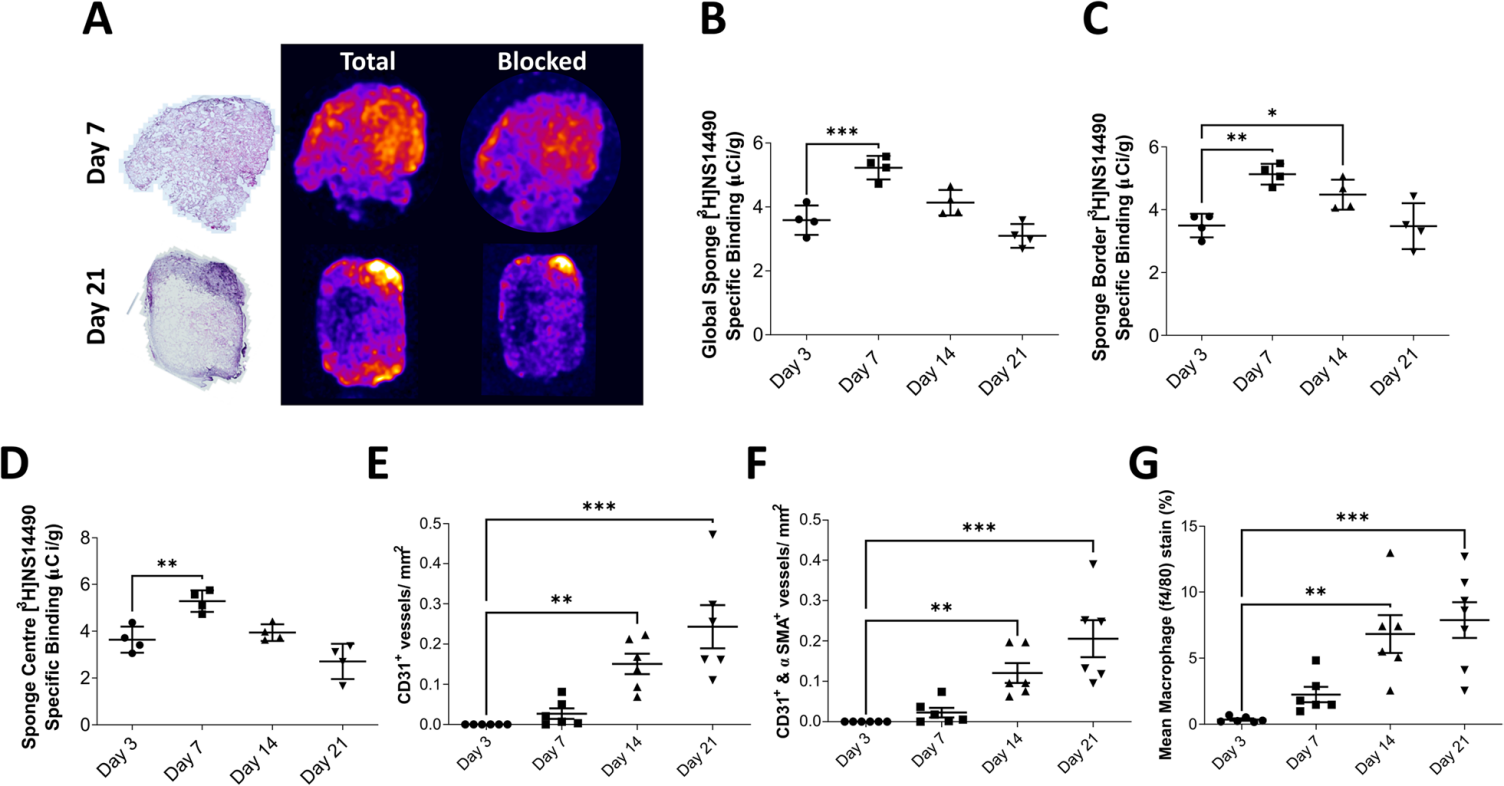
Characterization of α7nAChR in a murine inflammatory angiogenesis sponge implantation model reveals an early peak in expression corresponding with the beginning of angiogenesis and macrophage infiltration.** A** Representative H&E (left) and autoradiography performed with the α7nAChR selective radiotracer [^3^H]NS14490. The brighter colors represent areas of higher uptake. Specific binding was blocked using 10 µM NS14490 as shown in the blocking images. **B** Quantification of [^3^H]NS14490 specific binding across the whole sponge, **C** sponge border and **D** sponge center. *N* = 4, * = *P* ≤ 0.05, ** = *P* ≤ 0.01 and *** = *P* ≤ 0.001 using an ordinary one-way ANOVA with post hoc Dunnett’s versus day 3.** E** Histological quantification of sponge vasculature assessing CD31^+^ vessels and **F** CD31 + and αSMA + vessels. *N* = 6, ** = *P* ≤ 0.01 and *** = *P* ≤ *P* ≤ 0.001 using a Kruskal–Wallis test with post hoc Dunn’s versus day 3. **G** Quantification of f4/80 macrophage signal within the sponge, *N* = 6–7, ** = *P* ≤ 0.01 and *** = *P* ≤ 0.001 using an ordinary one-way ANOVA with post hoc Dunnett’s versus day 3

Following implantation of the sponge into the flank of the mice, the tissue was fixed, processed, and sectioned prior to [^3^H]NS14490 autoradiography. Specific binding of ^3^H-NS14490 across the whole sponge increased from 3.6 ± 0.2 µCi/g at day 3 post-implantation to 4.9 ± 0.2 µCi/g at day 7 (*P* < 0.01), followed by a reduction in specific binding at day 14 and 21 (Fig. 2A, B). As the sponges are vascularized inwards from the outer edge, quantification was also performed in the outer border (Fig. 2C) and center (Fig. 2D) which showed the same pattern in each region. This specific binding pattern matches the onset of vessel formation, but not peak blood vessel density which was day 21 (Fig. 2E, F). It also matches the initial infiltration of macrophages into the sponge, but again does not match the peak which was day 21 (Fig. 2G).

### Autoradiography with [^3^H]NS14490 following MI reveals a late infarct ɑ7nAChR signal from d14 which is strongly associated with extracellular matrix deposition

**Fig. 3:**
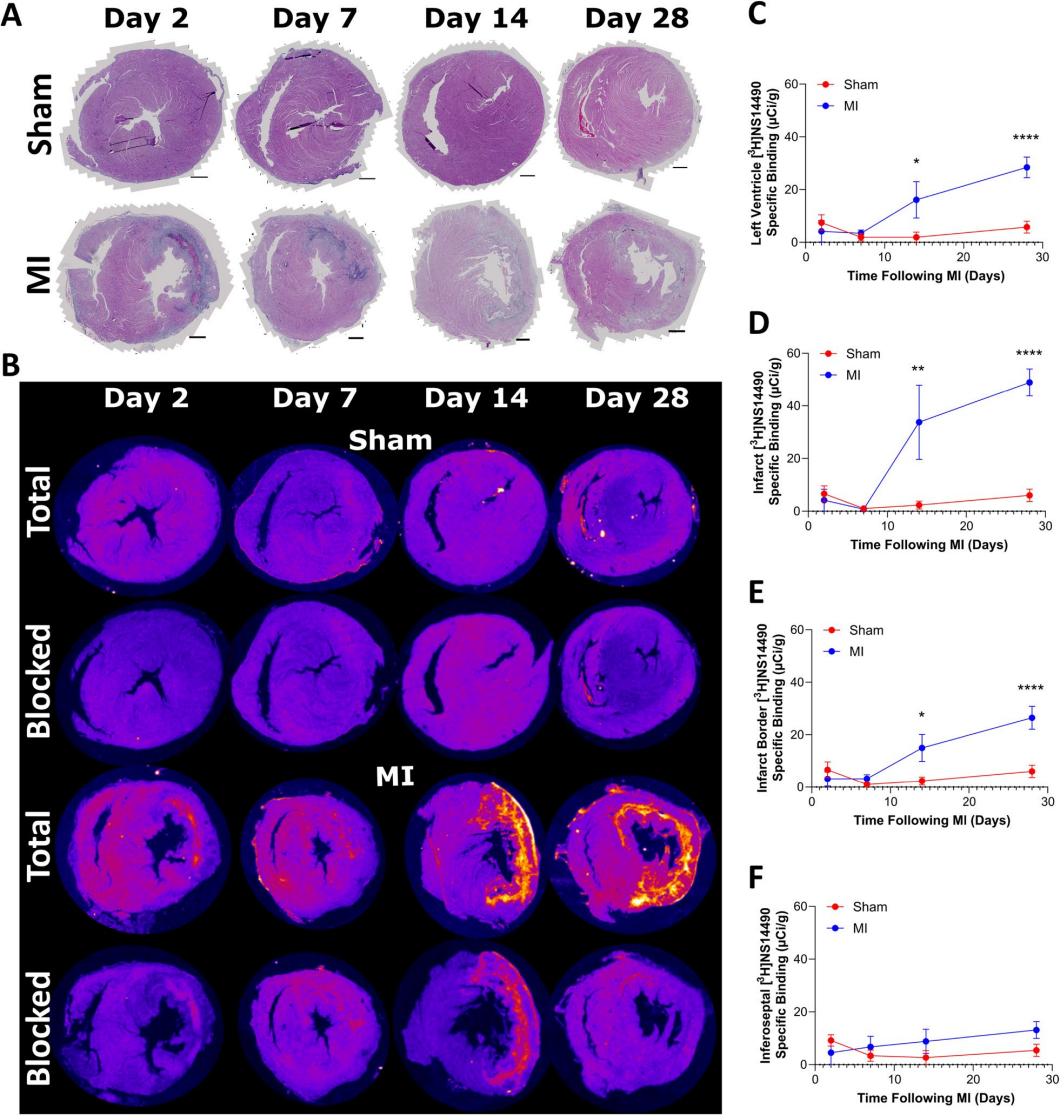
[^3^H]NS14490 autoradiography following MI reveals high α7nAChR expression within the infarct and border region at day 14 and day 28 following ischemia and reperfusion injury.** A** Representative H&E images of the heart from sham (non-infarcted) and MI animals from day 2 to day 28, scale bar = 1 mm. **B** Corresponding [^3^H]NS14490 autoradiography images showing total binding and blocked (10 µM NS14490) binding examples. **C** Quantification of the autoradiography specific binding signal across the left ventricle, with **D** showing quantification in the infarct, E infarct border and **F** remote inferoseptal region. *N* = 4–6, * = *P* ≤ 0.05, ** = *P* ≤ 0.01 and **** = *P* ≤ 0.0001 using two-way ANOVA with post hoc Šidák’s test for sham versus MI.

H&E staining in the rat cardiac tissue was used to draw regions of interest within the [^3^H]NS14490 autoradiography dataset (Fig. 3A). A low level of α7nAChR specific binding was found within the sham cohorts at all time points (1–6.6 µCi/g) (Fig. 3B). The α7nAChR signal was highest in the MI cohort, with [^3^H]NS14490 specific binding mainly localized to infarct from day 14 (infarct = 33.8 ± 14.1 µCi/g, *P* ≤ 0.01 versus sham), then peaking at day 28 (infarct = 48.9 ± 5.1 µCi/g, *P* ≤ 0.0001 versus sham, Fig. 3B–D). A similar trend was observed in the infarct border territory (Fig. 3E). No changes were observed between the sham group and the remote inferoseptal myocardium of the MI group (Fig. 3F).

**Fig. 4:**
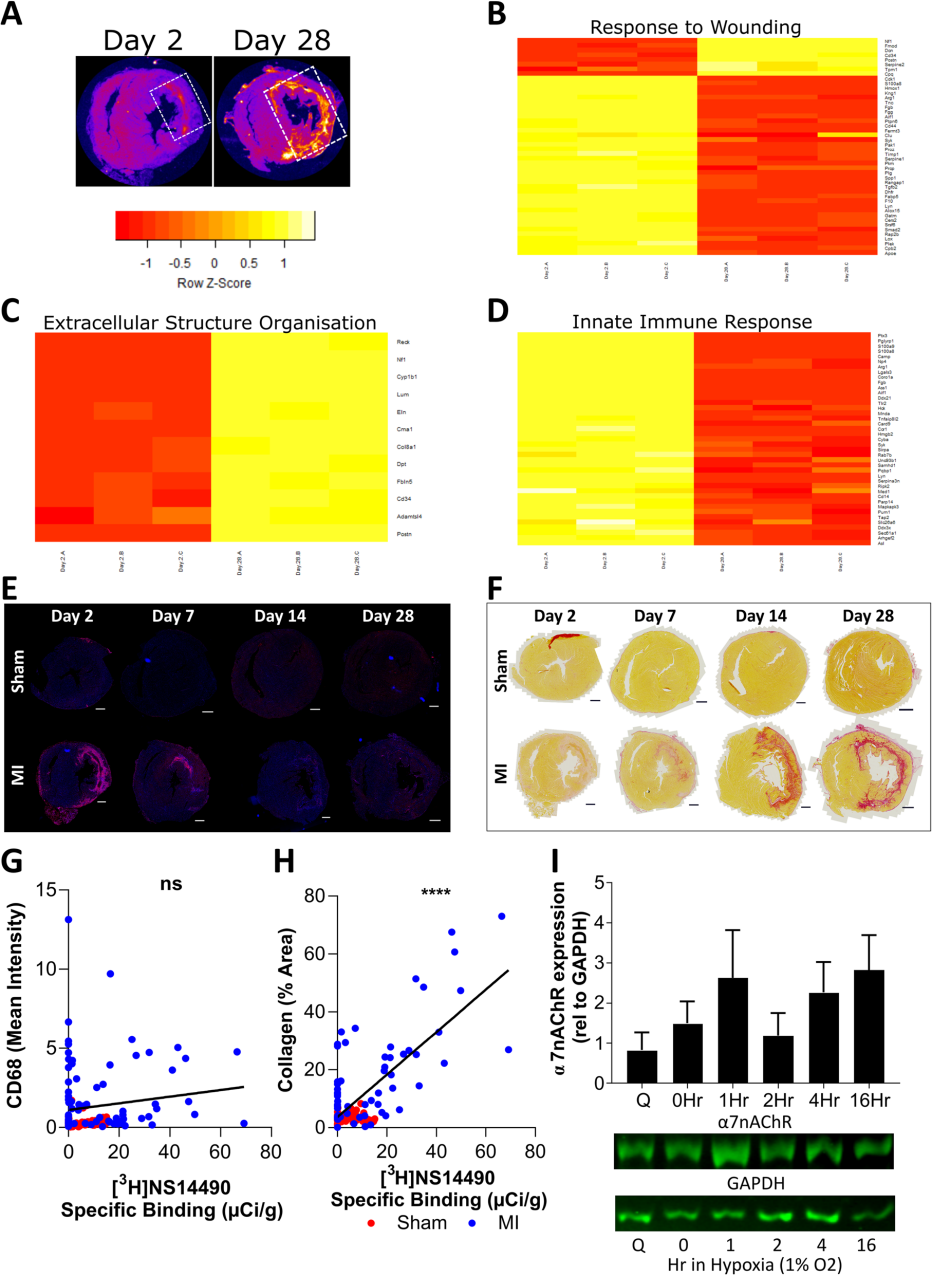
The α7nAChR signal correlates with changes in wound healing, specifically collagen deposition, and is expressed in rat cardiac fibroblasts. **A** White dashed box on NS14490 autoradiography images indicate example areas which were sampled for proteomics. **B** Proteomic heatmap of differentially expressed proteins within the infarct at day 2 (lanes 1–3) versus day 28 (lanes 4–6) which are associated with wound healing, **C** extracellular structure organization and **D** the innate immune response. Proteomics was performed in triplicate using pooled tissue from 4 to 6 rats. **E** Histological evaluation of the macrophage marker CD68 (red) which peaks at day 2 and **F** total collagen staining by Picro Sirius Red which peaks later. **G** The α7nAChR autoradiography signal does not correlate with CD68 but instead strongly correlates with **H** total collagen signal. The correlations graphs consist of global left ventricle, infarct or anterolateral equivalent, infarct border and remote or inferoseptal equivalent regions in 3–6 rats. NS = not significant and **** = *P* ≤ 0.0001 using Pearson correlation. **I** Western blot investigation of α7nAChR protein expression in rat cardiac fibroblasts during quiescent conditions (Q), normoxic proliferating conditions (0Hr) and exposure to 1% hypoxia (1–16 h) in proliferating conditions, *N* = 3

Proteomic profiling of α7nAChR expressing infarct tissue at day 28 versus day 2 infarct was carried out to understand more about the molecular mechanisms underpinning this expression pattern (Fig. 4A–D). This analysis revealed opposing changes across two distinct groups of proteins involved in the “response to wounding” (Fig. 4B). In addition, a group of proteins associated with “extracellular structure organization” was upregulated and a group associated with “innate immune response” was downregulated at day 28 versus day 2 (Fig. 4C, D). Guided by the results so far, and the fact α7nAChR expression was increased after the expected peak for an angiogenic-associated signal [30], further histological staining was performed for comparison to the [^3^H]NS14490 signal. CD68 staining for monocyte/macrophage detection revealed an early influx of CD68^+^ cells at day 2 which was resolved by day 14 (Fig. 4E). This marker did not did not correlate with α7nAChR expression (Fig. 4G). Picro Sirius red staining for total collagen revealed that collagen deposition within the infarct region increased from day 7 toward day 28 (Fig. 4F). This signal strongly correlated with the α7nAChR expression pattern (*P* =  < 0.0001, Fig. 4H), which was driven by the data points at day 14 and day 28 (Additional file 1: Fig. S2). In order to determine the involvement of cardiac fibroblasts in the collagen-associated ɑ7nAChR signal in the rat heart, rat cardiac fibroblasts were isolated and cultured. α7nAChR protein expression was assessed during normoxic and hypoxic conditions as performed with the humans cells in this study. Overall, these results show that rat cardiac fibroblasts express α7nAChR under both normoxic and hypoxic conditions (Fig. 4I, Additional file 1: Fig. S1). No statistically significant changes were observed between groups.

## Discussion

The mechanisms underlying cardiac wound healing remain poorly understood, and over time these processes influence the degree of adverse ventricular remodeling leading the heart into a state of failure [1, 2]. At present, there are limited noninvasive, holistic, imaging biomarkers which are capable of detecting and quantifying cardiac wound healing across the spectrum of processes which are driven by a coalition of different cell types [3]. Going into this study it, was clear that α7nAChR plays an important role in the regulation of inflammation [5, 12, 14] and stimulation of angiogenesis [4, 16, 17]. What was not clear was whether this target could serve as an imaging marker for cardiac repair and remodeling-associated processes. In order to address this gap, we set out to investigate which conditions were associated with α7nAChR expression, and how this changed over time during tissue remodeling and repair. In vitro assessment of macrophage and vascular cells revealed an endothelial cell specific increase in α7nAChR expression in response to hypoxia and proliferation, and the highest overall expression within monocyte-derived macrophages. Prior to assessment of α7nAChR following MI, this receptor’s relationship to inflammation and angiogenesis was assessed using the inflammation-driven angiogenic sponge implantation model. This revealed that the peak α7nAChR signal was associated with the onset of angiogenesis and macrophage accumulation, but did not match their respective peaks. Lastly, MI-driven left ventricular remodeling revealed no early signal coinciding with the onset of inflammation, or of angiogenesis, but instead an unexpected late infarct ɑ7nAChR signal that was strongly associated with extracellular matrix deposition. We then went onto demonstrate that rat cardiac fibroblasts express ɑ7nAChR which suggests these cells are the source of this target within the infarct.

At the beginning of this study, we hypothesized that ɑ7nAChR could serve as an imaging target for the assessment of angiogenesis and pro-reparative inflammation using NS14490. This hypothesis is supported by the rapid endothelial cell-specific increase in ɑ7nAChR expression in response to proliferation and hypoxia. Also, the fact that macrophages had the highest relative ɑ7nAChR expression supports the important role this receptor plays in the innate immune system. Further to this, the rapid increase in ɑ7nAChR expression (2 h post-exposure to hypoxia) and the early ɑ7nAChR signal in the sponge model suggest that this receptor could be acting as an initial upstream signal early in the cascade of responses required for tissue remodeling, rather than being required to sustain these responses. One of the main drivers of angiogenesis, VEGF, peaks between day 5–10 [31, 32], coinciding with our day 7 peak in ɑ7nAChR signal. Alternatively, in light of the fibroblast-associated ɑ7nAChR activity demonstrated in this paper, the high ɑ7nAChR signal within the first week of sponge implantation could also be explained by fibroblast activity which results in the total fibrovascular encapsulation of the sponge around this time point [32–34]. The period of fibrotic response occurs much earlier in the sponge than is seen within the rat heart following myocardial infarction, which could explain the differences observed in signal timelines. However, it should be noted that the inflammatory drivers of tissue remodeling in the sponge implantation model are very different to those which occur in response to infarction in the heart, and therefore it is reasonable to expect that ɑ7nAChR activity may differ across both models. In addition, both angiogenic and fibrotic-related activities may be contributing to the ɑ7nAChR signal in the sponge. Our rat MI data did not show an early increase in ɑ7nAChR signal associated with angiogenesis but, instead, demonstrated an unexpected late increase from d14 onwards. This signal strongly correlates with overall extracellular matrix deposition, shown by proteomics and collagen staining. Interestingly, infarct collagen deposition can be seen 1 week before the significant increase in ɑ7nAChR signal, suggesting that ɑ7nAChR could be associated with regulation of fibrosis rather than stimulation. We also demonstrated in vitro that rat cardiac fibroblasts express ɑ7nAChR under all conditions which we investigated, and others have demonstrated cardiac fibroblast ɑ7nAChR expression [13, 35]. It should be noted that these fibroblasts have heterogeneous ɑ-smooth muscle actin expression and are therefore inherently in an “activated” myofibroblast state [36]. Overall, there are very limited data on the role of ɑ7nAChR during fibrosis. In vivo, one study which used Donepezil to treat heart failure in rats post-MI demonstrated that peripheral blockage of ɑ7nAChR leads to increased fibrosis and adverse cardiac remodeling [6]. Outside of MI, the role of ɑ7nAChR during right ventricular fibrosis in an experimental rat pulmonary hypertension model has been recently investigated [35]. In this model, which uses an endothelial growth factor inhibitor and hypoxic chamber exposure, the authors demonstrate that right ventricular fibrosis and dysfunction are associated with increases in ɑ7nAChR expression in the right ventricle, but no changes are seen in the left ventricle. They go onto show that pharmacological antagonism of ɑ7nAChR reverses this trend and improves right ventricle function. In the same study, right ventricle tissue from pulmonary hypertension patients had increased collagen and acetylcholine (the endogenous ligand of ɑ7nAChR), but no increase in ɑ7nAChR was seen. While our MI findings do not support our original hypothesis that ɑ7nAChR could be a potential imaging target for angiogenesis and resolution of inflammation in the heart, taken together with the current literature on ɑ7nAChR in fibrosis, they suggest an alternative relationship between ɑ7nAChR and fibrosis which in itself is vital for cardiac repair and warrants further investigation in myocardial infarction.

While the final focus of this study was on ɑ7nAChR in the context of cardiac remodeling and repair, this receptor is also a potential imaging target in other remodeling processes throughout the body. In human carotid endarterectomy specimens, ɑ7nAChR staining has been found to localize with macrophages, T cells, endothelial cells and vascular smooth muscle cells [37]. In the same study, ablation of ɑ7nAChR in hematopoietic cells was shown to be pro-atherosclerotic by increasing inflammation. Since angiogenesis can be detrimental in atherosclerosis, it is not yet clear how ɑ7nAChR imaging in this context should be interpreted [5, 38]. Several preclinical studies have demonstrated the therapeutic potential of ɑ7nAChR stimulation following stroke [39–41]. While ɑ7nAChR imaging has not yet been included, one study utilized radiotracers for inflammation ([^18^F]DPA-714) and matrix metalloprotease (MMP) [41]. Here, they found that stimulation of ɑ7nAChR led to beneficial remodeling with reduced inflammatory and increased MMP PET signals in rats after transient middle cerebral artery occlusion. Stimulation of MMP, a set of enzymes that regulate extracellular matrix by the degradation of collagen, supports the possible role of ɑ7nAChR in the regulation of collagen by cardiac fibroblasts in our MI data.

The ɑ7nAChR radiotracer utilized in this tissue study is the tritium version of the ^18^F-labeled compound NS14490. NS14490 has been shown to have high affinity and selectivity for ɑ7nAChR, with a Ki of 2.5 nM which is 40.8 times higher than its affinity for ɑ2β4 and 400 times higher than ɑ4β2 nAChR subtypes [19]. The same study also demonstrated that it has a suitable metabolic profile in mice making it suitable for PET imaging. In another study, [^18^F]NS14490 was investigated in juvenile pigs which revealed a similar metabolic profile, and a binding potential (BPnd) of 0.5 calculated by blocking with the lower affinity ɑ7nAChR ligand NS6740 [20]. This study also demonstrated high uptake in the brain vasculature, highlighting the vascular expression of ɑ7nAChR. In addition, NS14490 is an agonist of ɑ7nAChR which theoretically means that use of this approach could be more selective for the available and activated state of this receptor. This could make NS14490 binding more reflective of receptor activity than an antagonist alternative. Overall, the properties of NS14490 make it a good candidate for further translation of this approach. Several ^18^F-labeled alternatives to NS14490 are also being developed for ɑ7nAChR PET imaging. [^18^F]YLF-DW, which has an ɑ7nAChR affinity similar to NS14490 [42], is also an agonist based radiotracer at the preclinical stage which has shown high uptake in carotid plaques of ApoE^−/−^ mice [43]. The ɑ7nAChR radiotracer [^18^F]ASEM has been shown to localize with atherosclerotic plaques in preclinical models [44], and is the most advanced option in terms of development as it has already undergone human trials [45]. However, its structure is based on an ɑ7nAChR antagonist rather than an agonist.

There are several limitations within this study. Firstly, the effect of macrophage polarization on ɑ7nAChR expression/signal was not investigated in our in vitro cell work, or by histology in our rodent sponge and MI models. However, based on the literature it is likely that ɑ7nAChR expression will be favored in an anti-inflammatory M2-like phenotype [12, 14]. The hypoxia strategy employed in this study is unlikely to have had an effect on the polarization of the initially Mφ-like macrophages based on previous evidence [46]. However, if these macrophages were previously primed toward an M1-like or M2-like phenotype, the overall effect of hypoxia would be polarization toward a more M2-like macrophage [46]. In contrast to this, intermittent cycling of hypoxia with normoxia has been shown to induce a more proinflammatory phenotype in initially Mφ-like and M1-like cells [47]. The second limitation of this work is that, while peripheral targets have been investigated for other ɑ7nAChR radiotracers, NS14490 has mainly been investigated in the brain. Therefore, the presence of radiometabolites in the heart have not been assessed and should be investigated in future studies. It should be noted though that the presence of radiometabolites in the brain are low, and radiometabolism in the plasma is slow [19]. While good levels of specific binding were achieved in this study using fixed tissue, there is a possibility this could be further improved by using unfixed tissue which should be explored in future research. Finally, the radiotracer aspects of this study were conducted in tissue by autoradiography rather than in vivo PET imaging. Future studies should validate these findings by noninvasive PET in a longitudinal manner, with the time points to be assessed guided by the results reported in this manuscript.

## Conclusions

In conclusion, this study demonstrates ɑ7nAChR expression in human macrophage and vascular cells, with the highest relative expression in macrophages but with only endothelial cells demonstrating a hypoxia-driven response. While the ɑ7nAChR signal pattern in the mouse sponge model indicates a peak expression early in the remodeling process, and aligning with the beginning of angiogenesis and macrophage accumulation, this is not the case in the rat MI model. Instead, ɑ7nAChR is expressed by rat cardiac fibroblasts and the autoradiography signal is strongly associated with extracellular matrix deposition and potentially regulation of fibrosis. Overall, these findings support the involvement of ɑ7nAChR across several processes central to cardiac remodeling and repair, with fibrosis most closely associated with ɑ7nAChR following MI, suggesting that this target could have utility as an imaging biomarker. Further studies are now required to assess this receptor in in vivo PET imaging studies which will allow the investigation of the relationship between ɑ7nAChR expression and cardiac function during adverse remodeling.

### Supplementary Information

**Additional file 1**. **Fig. S1**. Uncropped Western blot examples for all human and rat cell types used in this study. ɑ7nAChR is shown by the band at 57 kDa, and GAPDH is shown by the band at 38 kDa. Red text denotes the ladder positions. Blots were manually cut below the 49 kDa band following transfer and prior to the blocking step. **Fig. S2**. Total Collagen vs. α7nAChR autoradiography signal at individual time points in sham and MI cohorts. **A** Comparison of PSR quantification vs. [3H]NS14490 specific binding at day 2, **B** day 7, **C** day 14 and **D** day 28. The correlation graphs consist of global left ventricle, infarct or anterolateral equivalent, infarct border and remote or inferoseptal equivalent regions in 3-6 rats. *ns* not significant, **= *p* ≤0.01 and ****p ≤ 0.0001 using Pearson correlation

## Data Availability

The datasets used and/or analyzed during the current study are available from the corresponding author on reasonable request.

## References

[1] Prabhu SD, Frangogiannis NG (2016). The biological basis for cardiac repair after myocardial infarction. Circ Res.

[2] Chalise U, Becirovic-Agic M, Lindsey ML (2023). The cardiac wound healing response to myocardial infarction. WIREs Mech Dis.

[3] Tzahor E, Dimmeler S (2022). A coalition to heal—the impact of the cardiac microenvironment. Science.

[4] Heeschen C, Weis M, Aicher A, Dimmeler S, Cooke JP (2002). A novel angiogenic pathway mediated by non-neuronal nicotinic acetylcholine receptors. J Clin Invest.

[5] Vieira-Alves I, Coimbra-Campos LMC, Sancho M, da Silva RF, Cortes SF, Lemos VS (2020). Role of the α7 nicotinic acetylcholine receptor in the pathophysiology of atherosclerosis. Front Physiol.

[6] Li M, Zheng C, Kawada T, Inagaki M, Uemura K, Akiyama T (2021). Impact of peripheral α7-nicotinic acetylcholine receptors on cardioprotective effects of donepezil in chronic heart failure rats. Cardiovasc Drugs Ther..

[7] Hou Z, Zhou Y, Yang H, Liu Y, Mao X, Qin X (2018). Alpha7 nicotinic acetylcholine receptor activation protects against myocardial reperfusion injury through modulation of autophagy. Biochem Biophys Res Commun.

[8] Li H, Zhang ZZ, Zhan J, He XH, Song XM, Wang YL (2012). Protective effect of PNU-120596, a selective alpha7 nicotinic acetylcholine receptor-positive allosteric modulator, on myocardial ischemia-reperfusion injury in rats. J Cardiovasc Pharmacol.

[9] Noviello CM, Gharpure A, Mukhtasimova N, Cabuco R, Baxter L, Borek D (2021). Structure and gating mechanism of the α7 nicotinic acetylcholine receptor. Cell..

[10] Maouche K, Medjber K, Zahm JM, Delavoie F, Terryn C, Coraux C (2013). Contribution of α7 nicotinic receptor to airway epithelium dysfunction under nicotine exposure. Proc Natl Acad Sci U S A.

[11] Wada T, Naito M, Kenmochi H, Tsuneki H, Sasaoka T (2007). Chronic nicotine exposure enhances insulin-induced mitogenic signaling via up-regulation of α7 nicotinic receptors in isolated rat aortic smooth muscle cells. Endocrinology.

[12] Wang H, Yu M, Ochani M, Amella CA, Tanovic M, Susarla S (2003). Nicotinic acetylcholine receptor α7 subunit is an essential regulator of inflammation. Nature.

[13] Vang A, Clements RT, Chichger H, Kue N, Allawzi A, O’Connell K (2017). Effect of α7 nicotinic acetylcholine receptor activation on cardiac fibroblasts: a mechanism underlying RV fibrosis associated with cigarette smoke exposure. Am J Physiol Lung Cell Mol Physiol.

[14] Lee RH, Vazquez G (2013). Evidence for a prosurvival role of alpha-7 nicotinic acetylcholine receptor in alternatively (M2)-activated macrophages. Physiol Rep.

[15] Thackeray JT, Bengel FM (2018). Molecular imaging of myocardial inflammation with positron emission tomography post-ischemia: a determinant of subsequent remodeling or recovery. JACC Cardiovasc Imaging.

[16] Cooke JP (2007). Angiogenesis and the role of the endothelial nicotinic acetylcholine receptor. Life Sci..

[17] Yu JG, Song SW, Shu H, Fan SJ, Liu AJ, Liu C (2013). Baroreflex deficiency hampers angiogenesis after myocardial infarction via acetylcholine-α7-nicotinic ACh receptor in rats. Eur Heart J.

[18] Lee J, Cooke JP (2012). Nicotine and pathological angiogenesis. Life Sci.

[19] Rötering S, Scheunemann M, Fischer S, Hiller A, Peters D, Deuther-Conrad W (2013). Radiosynthesis and first evaluation in mice of [^18^F]NS14490 for molecular imaging of α7 nicotinic acetylcholine receptors. Bioorganic Med Chem.

[20] Rötering S, Deuther-Conrad W, Cumming P, Donat CK, Scheunemann M, Fischer S (2014). Imaging of α7 nicotinic acetylcholine receptors in brain and cerebral vasculature of juvenile pigs with [^18^F]NS14490. EJNMMI Res.

[21] Brilla CG, Zhou G, Matsubara L, Weber KT (1994). Collagen metabolism in cultured adult rat cardiac fibroblasts: Response to angiotensin II and aldosterone. J Mol Cell Cardiol.

[22] Gündüz D, Hamm CW, Aslam M (2017). Simultaneous isolation of high quality cardiomyocytes, endothelial cells, and fibroblasts from an adult rat heart. J Vis Exp.

[23] Melzer M, Beier D, Young PP, Saraswati S (2020). Isolation and characterization of adult cardiac fibroblasts and myofibroblasts. J Vis Exp..

[24] Wu J, Miller E, Davidson C, Walker BR, Hadoke PWF (2022). Enhanced Angiogenesis by 11βHSD1 Blockage Is Insufficient to Improve Reperfusion Following Hindlimb Ischaemia. Front Cardiovasc Med.

[25] Kim HJ, Na JI, Min BW, Na JY, Lee KH, Lee JH (2014). Evaluation of protein expression in housekeeping genes across multiple tissues in rats. Korean J Pathol.

[26] Wiśniewski JR, Mann M (2016). A proteomics approach to the protein normalization problem: Selection of unvarying proteins for MS-based proteomics and western blotting. J Proteome Res.

[27] Aguilan JT, Kulej K, Sidoli S (2020). Guide for protein fold change and: P-value calculation for non-experts in proteomics. Mol Omi R Soc Chem.

[28] Wang J, Vasaikar S, Shi Z, Greer M, Zhang B (2017). WebGestalt 2017: a more comprehensive, powerful, flexible and interactive gene set enrichment analysis toolkit. Nucleic Acids Res.

[29] Schwende H, Fitzke E, Ambs P, Dieter P (1996). Differences in the state of differentiation of THP-1 cells induced by phorbol ester and 1,25-dihydroxyvitamin D3. J Leukoc Biol.

[30] Zhao W, Zhao T, Chen Y, Ahokas RA, Sun Y (2009). Reactive oxygen species promote angiogenesis in the infarcted rat heart. Int J Exp Pathol.

[31] Marques SM, Campos PP, Castro PR, Cardoso CC, MôAND Ferreira, Andrade SP (2011). Genetic background determines mouse strain differences in inflammatory angiogenesis. Microvasc Res.

[32] Mendes JB, Campos PP, Ferreira MAND, Bakhle YS, Andrade SP (2007). Host response to sponge implants differs between subcutaneous and intraperitoneal sites in mice. J Biomed Mater Res Part B Appl Biomater.

[33] Small GR, Hadoke PWF, Sharif I, Dover AR, Armour D, Kenyon CJ (2005). Preventing local regeneration of qlucocorticoids by 11β-hydroxysteroid dehydrogenase type 1 enhances angiogenesis. Proc Natl Acad Sci U S A.

[34] Andrade SP, Fan TPD, Lewis GP (1987). Quantitative in-vivo studies on angiogenesis in a rat sponge model. Br J Exp Pathol.

[35] Vang A, Bos DD, Fernandez-Nicolas A, Zhang P, Morrison AR, Mancini TJ (2021). α7 Nicotinic acetylcholine receptor mediates right ventricular fibrosis and diastolic dysfunction in pulmonary hypertension. JCI Insight.

[36] Frangogiannis N (2000). Myofibroblasts in reperfused myocardial infarcts express the embryonic form of smooth muscle myosin heavy chain (SMemb). Cardiovasc Res.

[37] Johansson ME, Ulleryd MA, Bernardi A, Lundberg AM, Andersson A, Folkersen L (2014). α7 Nicotinic acetylcholine receptor is expressed in human atherosclerosis and inhibits disease in mice—brief report. Arterioscler Thromb Vasc Biol.

[38] MacAskill MG, Newby DE, Tavares AAS (2019). Frontiers in positron emission tomography imaging of the vulnerable atherosclerotic plaque. Cardiovasc Res.

[39] Han Z, Shen F, He Y, Degos V, Camus M, Maze M (2014). Activation of α-7 nicotinic acetylcholine receptor reduces ischemic stroke injury through reduction of pro-inflammatory macrophages and oxidative stress. PLoS One..

[40] Wang Y-Y, Lin S-Y, Chang C-Y, Wu C-C, Chen W-Y, Huang W-C (2023). α7 nicotinic acetylcholine receptor agonist improved brain injury and impaired glucose metabolism in a rat model of ischemic stroke. Metab Brain Dis.

[41] Aguado L, Joya A, Garbizu M, Plaza-García S, Iglesias L, Hernández MI (2023). Therapeutic effect of α 7 nicotinic receptor activation after ischemic stroke in rats. J Cereb Blood Flow Metab..

[42] Wang S, Fang Y, Wang H, Gao H, Jiang G, Liu J (2018). Design, synthesis and biological evaluation of 1,4-Diazobicylco[3.2.2]nonane derivatives as α7-Nicotinic acetylcholine receptor PET/CT imaging agents and agonists for Alzheimer’s disease. Eur J Med Chem.

[43] Wang D, Yao Y, Wang S, Zhang H, He ZX (2021). The availability of the α7-nicotinic acetylcholine receptor in early identification of vulnerable atherosclerotic plaques: a study using a novel 18F-label radioligand PET. Front Bioeng Biotechnol.

[44] Yang T, Wang D, Chen X, Liang Y, Guo F, Wu C (2021). 18F-ASEM imaging for evaluating atherosclerotic plaques linked to α7-nicotinic acetylcholine receptor. Front Bioeng Biotechnol.

[45] Hillmer AT, Li S, Zheng M-Q, Scheunemann M, Lin S, Nabulsi N (2017). PET imaging of α7 nicotinic acetylcholine receptors: a comparative study of [18F]ASEM and [18F]DBT-10 in nonhuman primates, and further evaluation of [18F]ASEM in humans. Eur J Nucl Med Mol Imaging.

[46] Ke X, Chen C, Song Y, Cai Q, Li J, Tang Y (2019). Hypoxia modifies the polarization of macrophages and their inflammatory microenvironment, and inhibits malignant behavior in cancer cells. Oncol Lett.

[47] Delprat V, Tellier C, Demazy C, Raes M, Feron O, Michiels C (2020). Cycling hypoxia promotes a pro-inflammatory phenotype in macrophages via JNK/p65 signaling pathway. Sci Rep.

