# Supplementary material for: Assessment of the alpha 7 nicotinic acetylcholine receptor as an imaging marker of cardiac repair-associated processes using NS14490
Source: EJNMMI Res. 2024 Jan 11;14:7. doi: 10.1186/s13550-023-01058-2 (PMC10784260; doi:10.1186/s13550-023-01058-2)
Supplement: Supplementary file 1 — Additional file 1. Fig. S1. Uncropped Western blot examples for all human and rat cell types used in this study. ɑ7nAChR is shown by the band at 57 kDa, and GAPDH is shown by the band at 38 kDa. Red text denotes the ladder positions. Blots were manually cut below the 49 kDa band following transfer and prior to the blocking step. Fig. S2. Total Collagen vs. α7nAChR autoradiography signal at individual time points in sham and MI cohorts. A Comparison of PSR quantification vs. [3H]NS14490 specific binding at day 2, B day 7, C day 14 and D day 28. The correlation graphs consist of global left ventricle, infarct or anterolateral equivalent, infarct border and remote or inferoseptal equivalent regions in 3-6 rats. ns not significant, **= p ≤0.01 and ****p ≤ 0.0001 using Pearson correlation [file 13550_2023_1058_MOESM1_ESM.docx]

**Supplementary**

**Title:**

Assessment of the alpha 7 nicotinic acetylcholine receptor as an imaging marker of cardiac repair-associated processes using NS14490

**Authors:**

Victoria JM Reid^1,2^, Wesley KX McLoughlin^1^, Kalyani Pandya^1,2^, Holly Stott^1^, Monika Iškauskienė ^3^, Algirdas Sackus^3^, Judit A Marti^4^, Dominic Kurian^4^, Thomas M Wishart^4^, Christophe Lucatelli^2^, Dan Peters^5,6^, Gillian A Gray^1^, Andrew H Baker^1^, David E Newby^1^, Patrick WF Hadoke^1^, Adriana AS Tavares^1,2^, Mark G MacAskill^1,2^*****.

*****Corresponding Author: Mark G MacAskill, [mark.macaskill@ed.ac.uk](mailto:mark.macaskill@ed.ac.uk), The University of Edinburgh, Edinburgh, UK.

1. Centre for Cardiovascular Science, The University of Edinburgh, Edinburgh, UK.

2. Edinburgh Imaging, The University of Edinburgh, Edinburgh, UK.

3. Chemistry Department, Kaunas University of Technology, Kaunas, Lithuania.

4. Proteomics and Metabolomics Facility, The Roslin Institute, University of Edinburgh, Edinburgh, UK.

5. DanPET AB, Malmo, Sweden.

6. Neurobiology Research Unit, Copenhagen University Hospital Rigshospitalet, Copenhagen, Denmark

**Keywords:**

Alpha 7 Nicotinic Acetylcholine Receptor, Imaging, Myocardial Infarction, Cardiac Repair


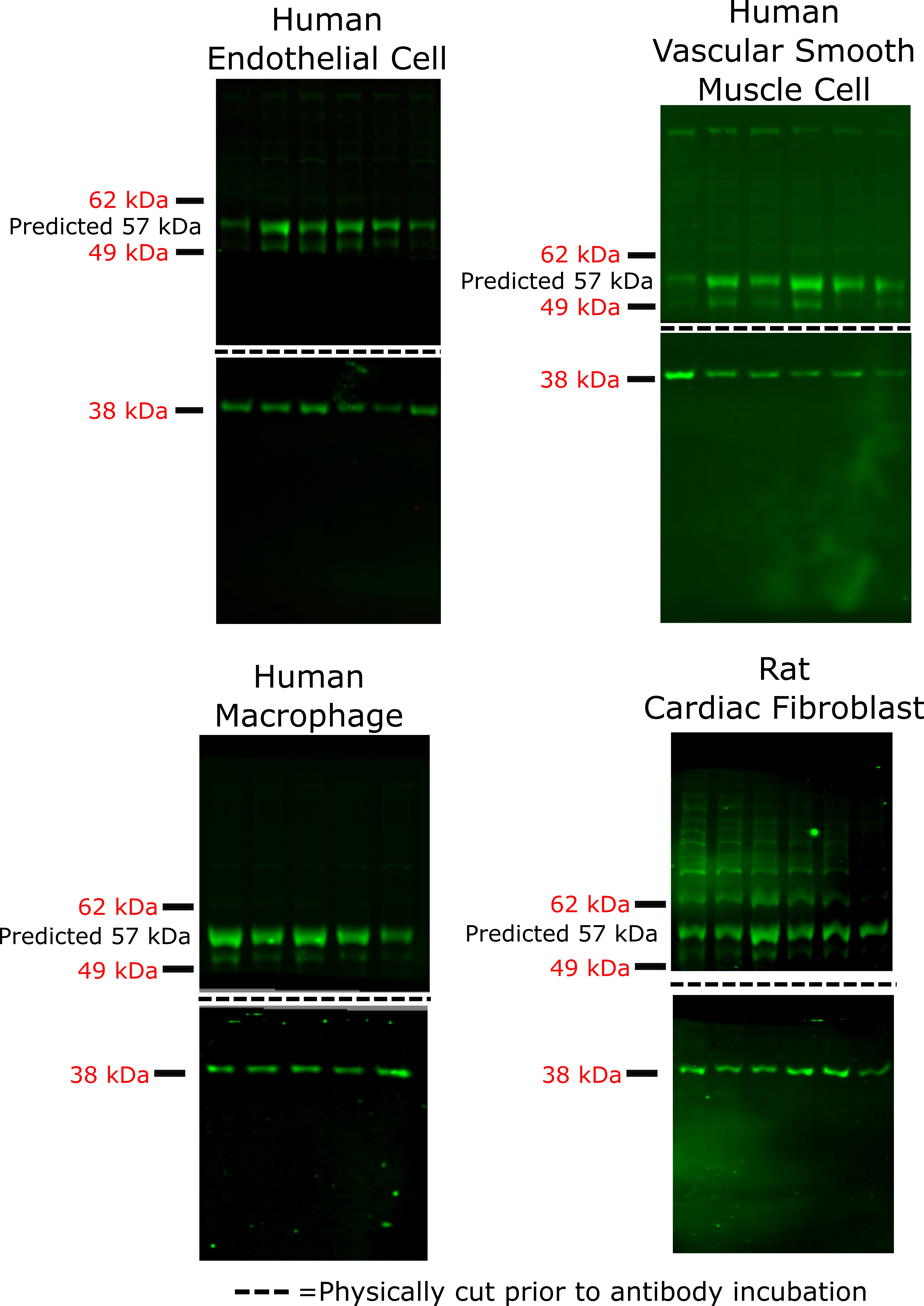


**Supplementary Figure 1. Uncropped Western blot examples for all human and rat cell types used in this study.** ɑ7nAChR is shown by the band at 57 kDa, and GAPDH is shown by the band at 38 kDa. Red text denotes the ladder positions. Blots were manually cut below the 49 kDa band following transfer and prior to the blocking step.


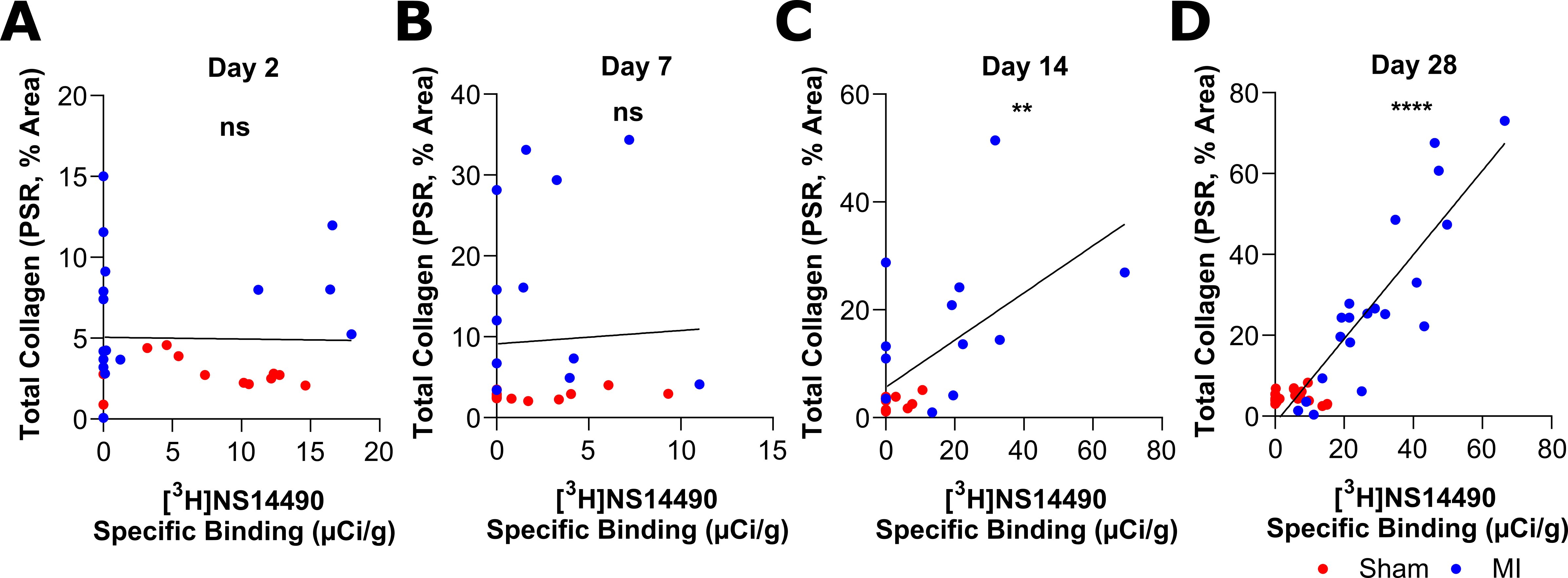


**Supplementary Figure 2. Total Collagen vs. α7nAChR autoradiography signal at individual time points in sham and MI cohorts. A)** Comparison of PSR quantification vs. [^3^H]NS14490 specific binding at Day 2, **B)** Day 7, **C)** Day 14 and **D)** Day 28. The correlation graphs consist of global left ventricle, infarct or anterolateral equivalent, infarct border and remote or inferoseptal equivalent regions in 3-6 rats. ns = not significant, **= p≤0.01 and ****= p≤0.0001 using Pearson correlation.
